# Successful completion of onchocerciasis elimination mapping (OEM) in Niger, West Africa

**DOI:** 10.1093/inthealth/ihad032

**Published:** 2023-05-15

**Authors:** Salissou Adamou, Daniel Boakye, Laouali Bouckari, Anne Heggen, Jamie Tallant, Yayé Youssouf, Charles D Mackenzie

**Affiliations:** Niger Programme Onchocercose et Filariose Lymphatique, Niamey, Niger; Reaching the Last Mile Fund, The END Fund, New York, NY 10016, USA; Noguchi Memorial Institute for Medical Research, Accra, Ghana; Niger Programme Onchocercose et Filariose Lymphatique, Niamey, Niger; Reaching the Last Mile Fund, The END Fund, New York, NY 10016, USA; Reaching the Last Mile Fund, The END Fund, New York, NY 10016, USA; Heller Keller International, Niamey, Niger; Reaching the Last Mile Fund, The END Fund, New York, NY 10016, USA; COR-NTD, Task Force for Global Health, Atlanta, GA 30030, USA

**Keywords:** elimination mapping, exclusion mapping, Niger, onchocerciasis

## Abstract

**Background:**

By 1987, onchocerciasis in Niger had been successfully controlled in the six endemic river basins. In 2017, onchocerciasis elimination mapping (OEM) was carried out to determine if there was any ongoing transmission in the country as a whole.

**Methods:**

The recommended OEM procedures were implemented.

**Results:**

Ten districts, that included 35 villages, required field investigation as sites of possible transmission. None of these were found capable of supporting black fly breeding, nor was there any evidence of the presence of *Simulium* sp. flies.

**Conclusions:**

The implementation of OEM indicates that there is no transmission of onchocerciasis currently taking place in these newly assessed sites in Niger.

## Introduction

The Niger Onchocerciasis Program began in 1976 under the umbrella of the West African Onchocerciasis Control Program (OCP), focusing on larvicidal vector control in areas where *Simulium* vectors were present in association with onchocercal disease.^1^ By 1987, onchocerciasis was regarded as having been successfully controlled in six river basins: Diamangou, Niger, Tapoa, Sirba, Mekrou and Goroubi. The program then shifted to surveillance by the Niger Ministry of Health. With the Global Onchocerciasis Program changing from disease control to infection elimination,^2,3^ there was a need to ensure that all the potentially endemic areas in the country were free of infection transmission. Thus the recommended procedure of onchocerciasis elimination mapping (OEM) was carried out to ensure national interruption of transmission.^4^

## Methods

A desk review was conducted in 2019 to produce an updated map of potential *Simulium* breeding sites based on the presence of suitable environmental characteristics and a review of historical documents relating to onchocerciasis. Districts were categorised as ‘unknown’ if they were adjacent to previously endemic districts, had never been mapped for this infection or had never received any form of control-related intervention for onchocerciasis. These districts were then subject to ‘ground truthing’ (GT) in the rainy season, where a trained program team capable of recognising *Simulium* sp. breeding sites interviewed key village informants in first-line villages and visited potential *Simulium damnosum* breeding sites.^4,5^ In addition, fly captures were conducted in locations where biting activity was reported. An assessment of collected findings was then made to determine the need for additional serological surveys for each district.^4^

## Results

Ten districts required further consideration through the OEM protocol: Ayerou, Tillaberi, Guallam, Bani Bangou, Abala and Filingue in the Tilaberi Region and Dosso, Doutchi, Loga and Tibiri in the Dosso Region (Table 1), with seven in need of GT. The Loga, Tibiri and Bani Bangeru health districts were excluded due to the lack of rivers capable of supporting vector breeding. GT and larval prospection failed to find breeding sites in any of the seven districts, and no black fly was ever captured during test captures in 36 villages (Table 1). All the biting activities reported were interpreted to be due to insects other than *Simuliidae*. A site in Figoune (Ayerou District) was found to be conducive to black fly development, however, no larvae were found there nor were there any reports of black flies being present.

**Table 1.** Field-based findings related to black fly breeding potential in locations suspected, after desk review, of having the potential of being breeding sites for black flies

Region	District	Presence of rivers possibly supporting breeding	Decision from desk review	Villages where interviews were carried out, n	Villages where vectors were caught in test captures, n	Villages where potential breeding sites were prospected, n	Sites where conditions were favourable for active breeding
Tillaberi	Ayerou	Yes	GT needed	2	0	2	1 site favourable^a^
	Tillaberi	No	No GT	NC	NC	NC	No
	Oualiam	Yes	GT needed	2	0	0	No
	Bani Bangou	No	No GT	NC	NC	NC	No
	Bankilaré	Yes	GT needed	16	0	11	No
	Filingue	Yes	GT needed	1	0	1	No
Dosso	Dosso	Yes	GT needed	10	0	10	No
	Loga	No	No GT	NC	NC	NC	No
	Tibiri	Yes	GT needed	2	0	2	No
	Doutchi	Yes	GT needed	2	0	2	No
Total				35	0	28	1^a^

NC: not carried out, as there were no rivers present.

^a^Although environmental conditions were favourable for supporting black fly breeding, no larvae or flies were found at this location.

The first two steps of the OEM investigation did not find any evidence of active black fly breeding in any of the ‘unknown’ locations, as they lacked suitable ecology to support the transmission of onchocerciasis. In the one location where the conditions appeared conducive, there was no evidence of the presence of this vector. Therefore, the third step in OEM, the serological assessment, was not required, as all sites filled criteria of being free of transmission.

The two major challenges faced in this study were the acquisition of historical information for the desk review and the difficulty of carrying out GT in areas where there were security issues—nevertheless all sites were eventually visited. Moving forward, a national post-elimination surveillance system will need to be developed to ensure there is no recrudescence of disease in the future. Any changes in the hydrology of the environment, such as deconstruction of the Kandadji Dam, that make the rivers more conducive to *Simulium* breeding need to be carefully monitored.

We suggest that countries seeking historical data for OEM or for dossier preparation should contact groups that were involved in either collecting or analysing data (country teams, supporting non-government organizations etc.); in African Programme for Onchocerciasis Control times, much of the assessment data were sent to the Regional Office for Africa in Ouagadougou, Burkina Faso. In addition, contacting individuals who have previously worked in a specific country, many of whom have retired, is a useful approach, as is searching the archives of scientific organisations where country presentations may have been made in the past (e.g. American Society of Tropical Medicine and Hygiene, Royal Society of Tropical Medicine and Hygiene etc).

## Conclusions

This implementation of OEM indicates that there is no transmission of onchocerciasis currently taking place in the newly assessed sites in Niger. It is vital that appropriate surveillance procedures^6^ are now implemented to confirm and ensure that no transmission remains.

## Supplementary Material

ihad032_Supplemental_File

## Data Availability

The data discussed in this article are available from the Niger Onchocerciasis Program, Ministry of Health, Niamey, Niger. Contact Dr Salissou Adamou. Email: sadamouba@yahoo.fr.

## References

[1] World Health Organization . Twenty years of onchocerciasis control in West Africa: review of the work of the Onchocerciasis Control Programme in West Africa from 1974 to 1994. Available from: https://apps.who.int/iris/handle/10665/275543 [accessed 23 April 2023].

[2] Traore MO, Sarr MD, Badji A et al. Proof-of-principle of onchocerciasis elimination with ivermectin treatment in endemic foci in Africa: final results of a study in Mali and Senegal. PLoS Negl Trop Dis. 2012;6(9):e1825. 23029586 10.1371/journal.pntd.0001825 PMC3441490

[3] Mackenzie CD, Homeida MM, Hopkins AD et al. Elimination of onchocerciasis from Africa: possible? Trends Parasitol 2012;28(1):16–22. 22079526 10.1016/j.pt.2011.10.003

[4] World Health Organization . Report of the Second Meeting of the WHO Onchocerciasis Technical Advisory Subgroup, Geneva, Switzerland, 12‒14 February 2018. Available from: https://www.who.int/publications/i/item/WHO-CDS-NTD-PCT-2018.11 [accessed 23 April 2023].

[5] Hamill L, Trotignon G, Mackenzie C et al. Navigating the way to onchocerciasis elimination: the feasibility and affordability of onchocerciasis elimination mapping. Int Health. 2022;14(Suppl 1):i17i23. 35169850 10.1093/inthealth/ihab083 PMC8986348

[6] World Health Organization . Guidelines for stopping mass drug administration and verifying elimination of human onchocerciasis. Criteria and procedures. Geneva: World Health Organization; 2016. 26913317

