# Supplementary figures and images for: Successful completion of onchocerciasis elimination mapping (OEM) in Niger, West Africa
Source: Int Health. 2023 May 15;16(2):227–9. doi: 10.1093/inthealth/ihad032 (PMC10911525; doi:10.1093/inthealth/ihad032)

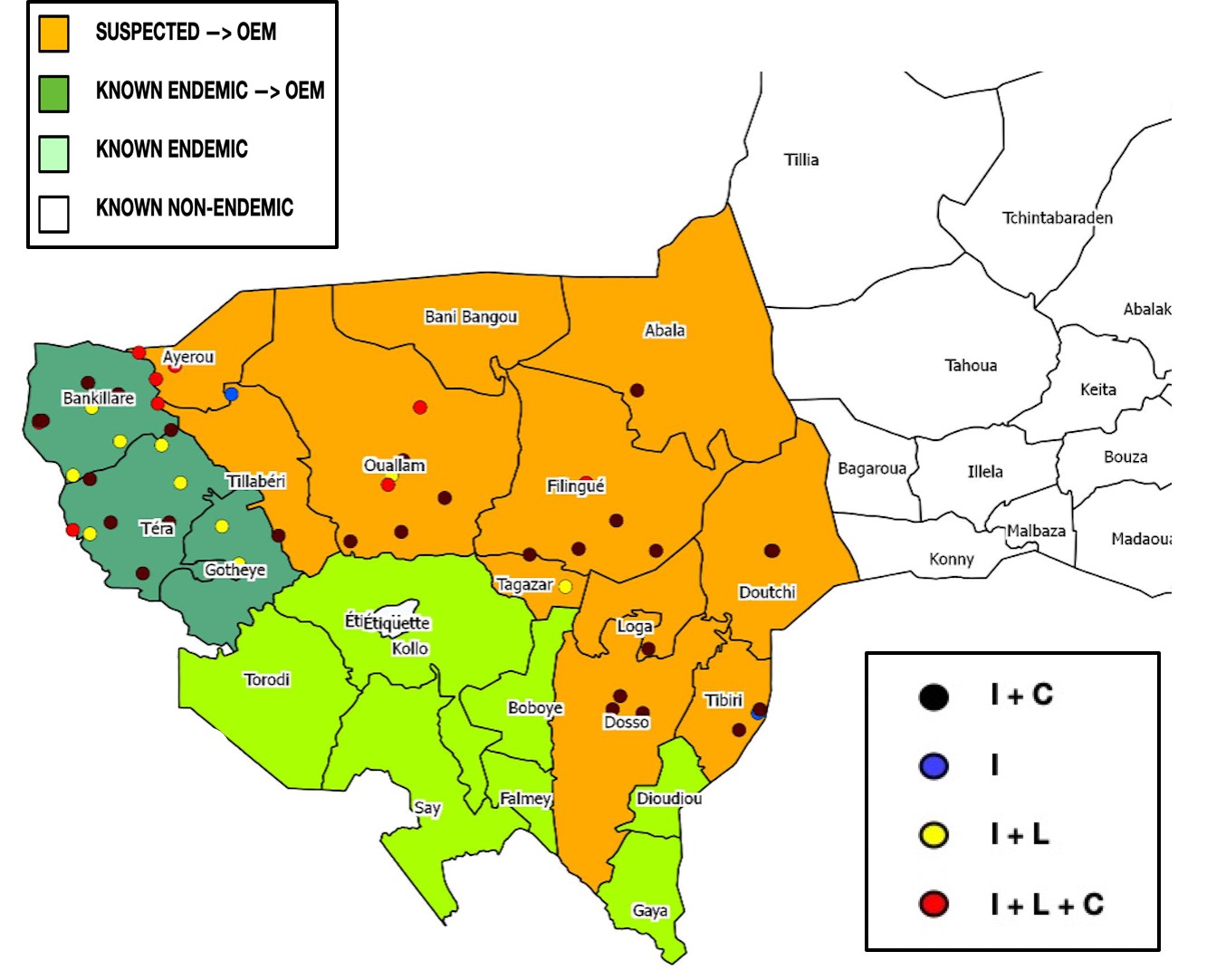

Supplement: ihad032_Supplemental_File [file ihad032_supplemental_file.jpeg]
